# MicroRNA in pancreatic adenocarcinoma: predictive/prognostic biomarkers or therapeutic targets?

**DOI:** 10.18632/oncotarget.4492

**Published:** 2015-07-16

**Authors:** Oronzo Brunetti, Antonio Russo, Aldo Scarpa, Daniele Santini, Michele Reni, Alessandro Bittoni, Amalia Azzariti, Giuseppe Aprile, Sabina Delcuratolo, Michele Signorile, Antonio Gnoni, Loredana Palermo, Vito Lorusso, Stefano Cascinu, Nicola Silvestris

**Affiliations:** ^1^ Medical Oncology Unit, National Cancer Research Centre, Istituto Tumori Giovanni Paolo II, Bari, Italy; ^2^ Department of Surgical, Oncological and Oral Sciences, Section of Medical Oncology, University of Palermo, Palermo, Italy; ^3^ Department of Pathology and Diagnostics, University of Verona, Verona, Italy; ^4^ Department of Medical Oncology, University Campus Bio-Medico, Rome, Italy; ^5^ Department of Medical Oncology, San Raffaele Scientific Institute, Milan, Italy; ^6^ AOU Ospedali Riuniti, Polytechnic University of the Marche Region, Ancona, Italy; ^7^ Clinical and Preclinical Pharmacology Laboratory, National Cancer Research Centre, Istituto Tumori Giovanni Paolo II, Bari, Italy; ^8^ Department of Medical Oncology, University Hospital of Udine, Udine, Italy; ^9^ Department of Medical Oncology, Hospital of Taranto, Taranto, Italy

**Keywords:** miRNAs, pancreatic adenocarcinoma, prognosis, biomarkers, therapy

## Abstract

Pancreatic ductal adenocarcinoma (PDAC) is a tumor with a poor prognosis, short overall survival and few chemotherapeutic choices. MicroRNAs (miRNAs) are non-coding, single-stranded RNAs of around 22 nucleotides involved in the pathogenic mechanisms of carcinogenesis and metastasis. They have been studied in many tumors in order to identify potential diagnostic, prognostic or therapeutic targets. In the current literature, many studies have analyzed the role of miRNAs in PDAC. In fact, the absence of appropriate biomarkers, the difficultly of early detection of this tumor, and the lack of effective chemotherapy in patients with unresectable disease have focused attention on miRNAs as new, interesting advance in this malignancy.

In this review we analyzed the role of miRNAs in PDAC in order to understand the mechanisms of action and the difference between the onco-miRNA and the tumor suppressor miRNA. We also reviewed all the data related to the use of these molecules as predictive as well as prognostic biomarkers in the course of the disease.

Finally, the possible therapeutic use of miRNAs or anti-miRNAs in PDAC is also discussed.

In conclusion, although there is still no clinical application for these molecules in PDAC, it is our opinion that the preclinical evidence of the role of specific miRNAs in carcinogenesis, the possibility of using miRNAs as diagnostic or prognostic biomarkers, and their potential therapeutic role, warrant future studies in PDAC.

## INTRODUCTION

Pancreatic ductal adenocarcinoma (PDAC) is one of the most aggressive cancers, with a 5-year survival of less than 5% [1]. Currently, PDAC causes an estimated 213, 000 deaths each year in the USA [2], because the majority of patients present with locally advanced or metastatic disease with only about 15% of patients candidate for surgical resection. However, even these patients experience relapse soon after surgery. In advanced stages or the relapse setting, new polychemotherapy combinations increase overall survival (OS) compared to gemcitabine (G) alone, in particular in fit patients, but life expectancy still remains unfavourable [3–5].

These data have led many researchers to consider the pathogenic mechanisms of carcinogenesis [6] and of PDAC in order to discover new potential diagnostic [7], prognostic [8] and therapeutic targets [9–10]. Furthermore the absence of appropriate biomarkers hinders both early detection and effective chemotherapy in patients with unresectable disease.

MicroRNAs (miRNAs) are small molecule endogenous non-coding RNA, single-stranded polymers formed from about 20–22 nucleotides, encoded by a nuclear DNA eukaryotic. They are mainly active in the transcriptional and post-transcriptional regulation of gene expression, binding to complementary sequences of target messenger RNA (mRNA) [11–13]. Identifying the specific binding of miRNAs has proved very difficult due to limited complementarity between miRNA and mRNA. The functional characterization of miRNAs depends greatly on the identification of binding partners of specific mRNA [14].

In normal tissues, the correct transcription, processing and binding of miRNAs for complementary sequences with specific mRNA involves the reduction or repression of target genes through a block in protein translation or stabilization of altered mRNA. The overall result is a normal rate of cell growth, proliferation, differentiation and cell death [15]. Since it was understood that miRNAs interfere with mRNAs that regulate the control of proliferation and apoptosis, it was hypothesized that these molecules could also play a role in the mechanisms of carcinogenesis [13].

The reduction or suppression of miRNAs that function as a tumor suppressor gene leads to the formation of tumors. A reduction or suppression of the levels of mature miRNA can occur because of defects in each phase of miRNA biogenesis, ranging from synthesis to uncoupling of the bases to a defect of exocytosis, and eventually leads to the inappropriate expression of the oncoprotein miRNA target. The overall result could lead to a greater proliferation, invasiveness or angiogenesis on the one hand, with a parallel decrease in the levels of apoptosis, undifferentiation or de-differentiation of a tissue, which ultimately leads to the formation of tumors [16–17]. The amplification or overexpression of a miRNA that has an oncogenic role could also contribute to the formation of tumors. In this situation, an increase in the amount of miRNA, which could be produced at inappropriate times or in the wrong tissues, reduces the brakes of the tumor suppressor gene and results in the amplification of the signals of cellular proliferation and then cancer progression. Increased levels of mature miRNAs may occur due to amplification of the genes of miRNAs, or to the presence of mutations because of a constitutively active promoter, greater efficiency in processing miRNA, or increased stability of the miRNA [18–19].

There are currently numerous miRNAs being studied within carcinogenesis in solid tumors [20–22] and hematologic malignances [23] and also in PDAC. A large amount of data has been presented and literature on the argument has increased dramatically in the last few years. Suppression or upregulation of miRNAs are clearly implicated in pancreatic cell proliferation, survival, invasion and metastases. Thus miRNA are promising discoveries for predictive/prognostic biomarkers or therapeutic targets.

In the present review we analyze the preclinical evidence for the role of specific miRNAs in pancreatic carcinogenesis, the possibility of using miRNAs as diagnostic or prognostic biomarkers in histological samples of PDAC or predictive molecules in the serum and pancreatic cyst fluid of patients who may develop a PDAC, evaluation of response/failure of cancer chemotherapy, and their potential therapeutic role.

## MiRNA IN PDAC: PRECLINICAL STUDIES

**Figure 1:**
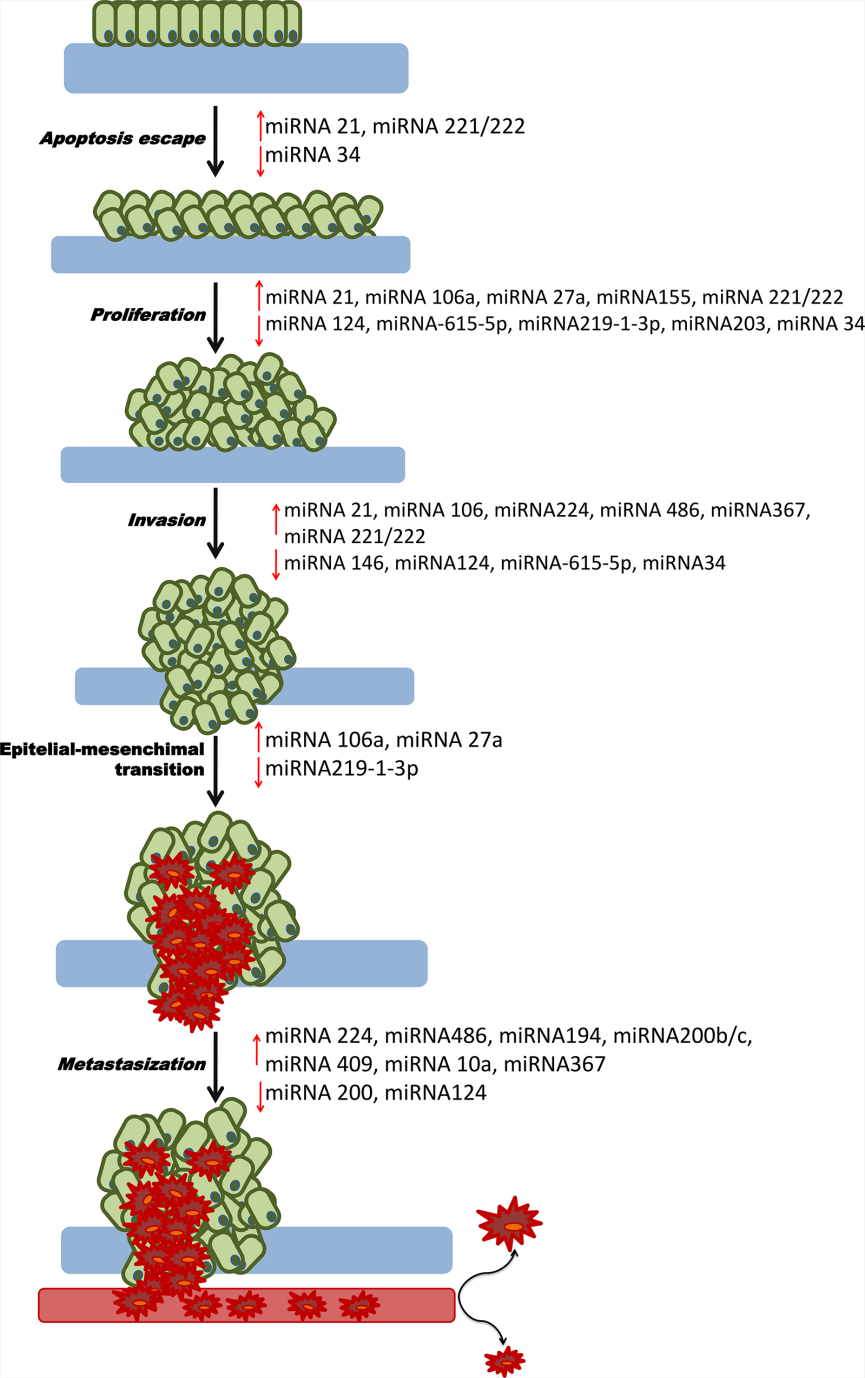
Role of miRNAs in PDAC carcinogenesis This picture represents all the mechanisms of carcinogenesis from the escape of apoptosis to metastasis. For each process, the down-regulated tumor suppressor miRNAs or upregulated oncogenes that may be the cause of PDAC are shown.

In order to understand the pathogenesis of cancer and the role of miRNAs in this malignancy, many authors, based on clinical evidence of aberrant miRNA production, developed cellular models and *in vivo* experiments to try to understand the deregulated mechanisms and the altered pathway at the root of carcinogenesis, metastasis and chemoresistance. To date many mechanisms and different levels of action of miRNAs in PDAC have been identified. Table 1 and Figure 1 are a summary of significant miRNAs and their relative role in PDAC carcinogenesis.

**Table 1:** A summary of significant up- and down-regulated miRNAs and their relative role in PDAC carcinogenesis

miRNA	Expression status	Target genes	Role in carcinogenesis	Reference
miRNA-21	Upregulation	Bcl-2	Promotion of cell proliferation, invasion, chemoresistance, escape from apoptosis	24–26
miRNA-34	Upregulation	Notch 1/2 Bcl-2	Escape from apoptosis, cell proliferation, invasion	27
miRNA-155	Upregulation	TP53INP1	Promotion of tumor development	28
miRNA-106a	Upregulation	TIMP-2	Promotion of cell proliferation, epitelial-mesenchymal transition, invasion	29
miRNA-27a	Upregulation	Spry-2	Promotion of PDAC cell growth, colony formation, migration	30
miRNA-221/222	Upregulation	MMP-2, MMP-9	Promotion of PDAC cell invasion	31
miRNA-224	Upregulation	CD40	Highly invasive PDAC, metastatic phenotype	32
miRNA-486	Upregulation	CD40	Highly invasive PDAC, metastatic phenotype	32
miRNA-194	Upregulation	EP300	Highly metastatic phenotype	33
miRNA-200b	Upregulation	EP300	Highly metastatic phenotype	33
miRNA-200C	Upregulation	EP300	Highly metastatic phenotype	33
miRNA-429	Upregulation	EP300	Highly metastatic phenotype	33
miRNA-10a	Upregulation	HOXA1	Promotion of metastatic phenotype	34
miRNA-367	Upregulation	Down-regulation of Smad7	Promotion of epitelial-mesenchymal transition, invasion, metastasis	35
miRNA-124	Downregulation	Rac-1	Promotion of cell proliferation, invasion, metastasis	36
miRNA-615-5p	Downregulation	IGF2, JUNB	Promotion of cell proliferation, migration, invasion	37–38
miRNA-200	Downregulation	Sox2, ZEB1, ZEB2	Promotion of metastatic phenotype, cell stemness	40–41
miRNA-219-1-3p	Downregulation	MUC4, cyclin D1, AKT-ERK pathway	Promotion of cell proliferation, cell migration	42
miRNA-203	Downregulation	Survivin	Promotion of tumor growth	43
miRNA-146a	Downregulation	IRAK-1	Promotion of invasion	44
miRNA-17-92	Downregulation	NODAL/ACTIVIN/TGF-β1	Chemoresistence	45

As already described, pathogenesis begins with the up-regulation of miRNA and deregulation of oncogenes or tumor suppressor miRNA.

### Onco-miRNA

The up-regulation of many miRNAs can lead to escape mechanisms from apoptosis, cell proliferation, invasion, metastasis and chemoresistence. In this perspective, miRNA-21 is the most studied molecule in all tumors. Starting from the over-expression of miRNA-21 in PDAC cell lines compared with non-malignant cells, Moriyama et al [24] demonstrated that cancer cells, transfected with miRNA-21, showed a significantly increased proliferation, matrigel invasion, and chemoresistance to G compared with non-transfected cells. Conversely, inhibition of miRNA-21 decreased proliferation, matrigel invasion, and chemoresistance to G. MiRNA-21 has also been studied for its anti-apoptotic mechanisms. In particular, Mace et al reported that hypoxia induces the expression of miRNA-21 in PDAC cells via HIF-1α upregulation and that miRNA-21 overexpression in a hypoxic microenvironment allows cells to avoid apoptosis [25]. Overexpression of miRNA-21 seems to directly induce upregulation of Bcl-2, resulting in a block of apoptosis, proliferation and chemoresistance of PDAC cell lines [26].

MiRNA-155 represses the expression of the pro-apoptotic gene *TP53INP1*, targeting its mRNA. Gironella et al demonstrated through immunohistochemical analysis in a nude mice model that TP53INP1 levels in PDAC are repressed by miRNA-155 and that restoration of *TP53INP1* expression inhibited tumor development [27].

Another upregulated miRNA is miRNA-106a [28]. Recently, Pei Li et al showed that expression levels of miRNA-106a were detected from 21 human PDAC samples using quantitative RT-PCR. The expression of miRNA-106a was significantly higher in PDAC tissues compared to adjacent normal pancreatic tissues. Based on this clinical evidence they studied the role of miRNA-106a in promoting cell proliferation that was proved by transfecting cell lines with miRNA-106a mimics; conversely miRNA-106a inhibitors were able to inhibit cell growth. Furthermore this study clarified the role of miRNA-106a in pancreatic tumorigenesis by promoting cancer cell proliferation, epithelial–mesenchymal transition and invasion by targeting tissue inhibitors of metalloproteinase 2 (TIMP-2). Regarding growth, colony formation and migration of PDAC cells, miRNA-27a targets the Spry2 protein, an antagonist of Ras/MAPK signaling. Overexpression of this miRNA inhibits Spry2, so Ras signaling and tumor growth could be enhanced. Transfection with a miRNA-27a inhibitor could upregulate the protein and reduces the growth [29]. More recently, in an *in vitro* study, miRNA-221/222 also resulted overexpressed in PDAC. MiRNA-221/222 overexpression significantly promoted growth and invasion, inhibiting apoptosis. PDAC cells transfected with this miRNA increased expression of the matrix metalloproteinases (MMPs) MMP-2 and MMP-9 [30].

Many miRNA are involved in metastatic phenotypes. MiRNA-224 and miRNA-486 were found to be involved in the progression of PDAC in murine orthotopic models by Mees et al [31] who studied 16 human PDAC cell lines and assessed local invasion and metastatic spread. Later, the same authors provided additional interesting preclinical evidence in murine orthotopic PDAC models, showing that overexpressed miRNA-194, miRNA-200b, miRNA-200c and miRNA-429 corresponded to reduce tumor suppressor gene EP300 mRNA and protein in highly metastatic PDAC cell lines compared with a non-metastatic or marginally metastatic phenotype [32]. MiRNA-10a overexpression also seems to promote metastatic behavior of PDAC cells. Retinoic acid receptor antagonists have been suggested as molecules potentially able to repress miRNA-10a expression and block metastasis. The antimetastatic activity can be prevented by the specific knockout HOX genes HOXB1 and HOXB3. Interestingly, the suppression of HOXB1 and HOXB3 in PDAC cells promotes the formation of metastases [33]. Regarding metastatic behavior, in an *in vitro* study miRNA-367 downregulated Smad7 expression promoting epithelial-to-mesenchymal transition by increasing TGF-β-induced transcriptional activity in PANC-1 and BxPC3 cells. Moreover, in male NOD/SCID mice, miRNA-367 promoted pancreatic cancer invasion and metastasis [34].

As with many well-known oncogenes, upregulation appears to play a key role in oncogenesis also with onco-miRNA. These preclinical studies on pathogenesis, however, need to understand the diagnostic, prognostic and therapeutic role of onco-miRNA.

### Tumor suppressor miRNA

In order to evaluate the down-regulation of miRNAs, the inactivation by hypermethylation of the DNA region encoding for miRNA could be one of the mechanisms that leads to repression of miRNAs. For example, hypermethylation mediates the silencing of miRNA-124 genes (including miRNA-124-1, miRNA-124-2 and miRNA-124-3), which are highly methylated in PDAC tissues as compared to non-cancerous tissues. MiRNA-124 seems to inhibit cell proliferation, invasion and metastasis and its downregulation is associated with worse survival in PDAC patients. The role of miRNA-124 as a tumor suppressor has been recently investigated by Wang et al who identified Rac1, a putative tumor promoter in PDAC, as a direct target of miRNA-124. MiRNA-124 acts by downregulating Rac1, leading to the inactivation of the MKK4-JNK-c-Jun pathway [35]. Another miRNA abnormally downregulated in PDAC cells through hypermethylation in its promoter region is miRNA-615 -5p. This molecule directly targets IGF2 and other genes, such as the proto-oncogene JUNB; moreover, it could interfere with the insulin signaling pathway, inhibiting pancreatic cancer cell proliferation, migration and invasion. Hypermethylation limits its inhibitory activity, contributing to tumor growth and the spread of metastasis [36]. More recently, Sun et al demonstrated that miRNA-615-5p targets mRNA coding for AKT2, repressing its expression. This miRNA inhibits PDAC cell proliferation, migration, and invasion promoting apoptosis in PANC-1 and MIA PaCa-2, and reduces tumor growth and the metastatic process in subcutaneous PDAC cells injected into male BALB/c nude mice. Moreover, miRNA-615 -5p expression in patient tissue was significantly lower in PDAC than in adjacent normal tissues [37]. DNA hypermethylation of the miRNA-148a region results in miRNA repression not only in PDAC samples but also in preneoplastic pancreatic intraepithelial neoplasia (Pan IN) lesions [38].

Regarding antiapoptotic molecules, miRNA-34 encompasses an important family of miRNAs implicated in cancer initiation and progression through their influence on the expression of genes and proteins that regulate cell proliferation and/or cell death. In fact upregulation of this miRNA family is induced by p53, which is inactivated in a large proportion of PDAC and has as targets stemness inducing Notch 1/2 and the anti-apoptotic Bcl-2. Ji et al examined the roles of miRNA-34 in p53-mutant human PDAC cell lines and the potential link to PDAC stem cells. Restoration of miRNA-34 expression causes downregulation of Bcl-2 and Notch1/2 and, in addition to inducing apoptosis, inhibits clonogenic cell growth and invasion [39].

Metastatic phenotype and cell stemness are strongly influenced by miRNA-200 family members by their suppressive activity. These molecules regulate negatively epithelial-to-mesenchymal transition (EMT) by suppressing stem cell factors such as Sox2 and inhibiting ZEB1 and ZEB2, negative regulators of E-cadherin (E-cad), which results upregulated; if these miRNAs are downregulated, E-cad is overexpressed and EMT results enhanced [40–41].

Among miRNAs with tumor-suppressor activity in PDAC, miRNA-219-1-3p has recently been shown to inhibit the oncogenic mucin MUC4, largely involved in PDAC carcinogenesis. MiRNA-219-1-3p expression is downregulated both in PDAC-derived cell lines and human PDAC tissues compared with normal counterparts. In an analysis of Lahdaoui et al, miRNA-219-1-3p overexpression induced a decrease of cell proliferation associated with a decrease of cyclin D1 and a deactivation of the Akt and Erk pathway, as well as a decrease of cell migration. Moreover, miRNA-219-1-3p injection in xenografted pancreatic tumors in mice decreased both tumor growth and MUC4 mucin expression [42]. Xu et al demonstrated that survivin is a direct target of miRNA-203 and that the protein levels of survivin negatively correlate with miRNA-203 levels in PDAC cell lines. The downregulation of survivin by transfection with miRNA-203 mimic in the PDAC cell line CFPAC-1 inhibited tumor growth [43]. A lower expression level of miRNA-146a in PDAC cells compared with non-malignant cells was shown by Li et al in 2010 [44]. In this study, the restoration of miRNA-146a suppressed invasion and was concomitant with a downregulation of EGFR and NF-kB regulatory kinase interleukin 1 receptor-associated kinase 1 (IRAK-1). More recently, the miRNA-17-92 cluster resulted downregulated in chemoresistant PDAC Cancer Stem Cells (CSCs) versus non-CSCs. Nevertheless, overexpression of this cluster reduces CSC self-renewal capacity *in vitro*. Moreover, tumourigenicity and chemoresistance are reduced in subcutaneous CSC-injected female NU-Foxn1nu nude mice, through targeting of multiple NODAL/ACTIVIN/TGF-β1 signalling cascade members with inhibition of the downstream targets p21, p57 and TBX3. As a consequence, the miRNA-17-92 cluster is a family of miRNAs with a putative potential of developing modulators in CSCs to overcome drug resistance in pancreatic CSCs [45].

The down-regulation of tumor suppressor miRNAs involves the activation of the growth, invasion and neoplastic transformation pathway. As a consequence, the restoration of these miRNAs through delivery systems will be one of the therapeutic strategies proposed in PDAC.

## MiRNA FOR DIAGNOSIS OF PANCREATIC ADENOCARCINOMA

Histological assessment of a suspicious mass within the pancreas is often performed by a biopsy of the primitive mass or its metastases. However, this biopsy may be difficult to interpret due to the small amount of tissue obtained and inflammatory changes which could complicate the distinction between malign and benign conditions.

MiRNAs can be detected in a very small amount of material and in altered samples. Furthermore, they show a high stability in tissues and fluids. For the first time in 2007 Bloomston et al showed that PDAC might have a distinct miRNA expression pattern useful to differentiate it from normal pancreas (NP) and chronic pancreatitis (CP). In particular, the authors analyzed over 1100 miRNA probes in microdissected paraffin blocks of NP, CP and PDAC and identified that 21 increased level miRNAs and 4 decrease level miRNAs correctly differentiated PDAC from NP tissue in 90% of samples. Moreover, 15 increased miRNA and 8 decreased miRNAs differentiated PDAC from CP with 93% accuracy [46]. Yue et al performed an analysis of miRNA expression from archived formalin-fixed paraffin embedded (FFPE) pancreatic resection specimens that showed how, compared to the normal pancreatic parenchyma, miRNA-148a and miRNA-217 expression levels were downregulated in PanIN, particularly in PanIN II–III and PDAC, whereas the level of miRNA-196 was significantly up-regulated in PDAC such as in PanIN II–III. In addition, miRNA-21 was significantly overexpressed in PDAC, and miRNA-10b was highly expressed in PanIN II–III [47].

Schultz et al analyzed the potential role of miRNAs as diagnostic cancer biomarkers in tissues from 170 PDAC and 107 ampullary adenocarcinomas. They evaluated the expression of 664 microRNAs, comparing results obtained with CP, NP and adenocarcinoma. Eighty-four miRNAs resulted differentially expressed between PDAC and NP. In fact, forty-three microRNAs showed higher levels and 41 resulted downregulated in PDAC; moreover 17 miRNAs were found to be higher and 15 reduced compared with CP. In this study a diagnostic classifier using 19 microRNAs was constructed with a sensitivity of 98.5% and a positive predictive value of 97.8% with an accuracy of 97.0%. This panel could therefore be useful in discriminating pancreatic and ampullary adenocarcinomas from CP and NP [48].

Recently, Brand et al have proposed another useful panel evaluating miRNA levels by relative quantitative polymerase chain reaction in 95 formalin-fixed paraffin-embedded specimens and 228 tissue samples collected by pancreatic biopsy, performed to define the nature of suspected solid masses. A 5-miRNA expression classifier, including miRNA-24, miRNA-130B, miRNA-135B, miRNA-148A, and miRNA-196, was developed and was able to identify PDAC. Detection of PDAC in EUS-FNA samples increased from 78.8% by cytology analysis alone to 90.8% when combined with miRNA analysis. In this series 22 additional PDAC cases were diagnosed among 39 samples initially classified as benign, indeterminate, or nondiagnostic by cytology alone [49].

These findings represent a step forward in obtaining a diagnosis of PDAC from the miRNA extracted from the biopsy of the lesion. However, more studies are required to better define a standard and complete panel of these biomarkers useful for diagnosis of a PDAC.

In this regard, miRNA plasma profiling has been explored as a minimally invasive procedure for detection of PDAC. Wang et al profiled four miRNAs, miRNA-21, miRNA-210, miRNA-155, and miRNA-196 from heparin-treated blood samples collected in 49 PDAC patients and 36 healthy controls. They reported that the miRNA profile in the plasma of these four miRNAs differentiated PDAC patients from healthy controls with a sensitivity of 64% and a specificity of 89% [50].

Liu J et al evaluated the level of seven miRNAs (miRNA-16, 21, 155, 181a, 181b, 196a and 210) in the plasma of 140 PDAC patients, 111 CP patients and 68 healthy controls, using real-time PCR. All the miRNAs considered were significantly aberrantly upregulated in the PDAC group compared with both the CP and healthy controls. Moreover, the combination of miRNA-16, miRNA-196a and CA19-9 was more effective in discriminating PDAC from non-PDAC and PDAC from CP, compared with the miRNA panel (miRNA-16 + miRNA-196a) or CA19-9 alone [51].

Morimura et al reported the diagnostic value of circulating miRNA-18a comparing plasma results from 36 PDAC patients and 30 healthy volunteers. Plasma concentrations of miRNA-18a were significantly higher in PDAC patients than in controls (*P* < 0.0001). Interestingly, plasma levels of miRNA-18a in patients with PDAC were significantly lower in postoperative samples than in preoperative samples (*P* = 0.0077) [52]. In this study, miRNA-18a levels in primary PDAC tissues paralleled those observed in plasma. Kawaguchi et al hypothesized a role for plasma miRNA-221 as a diagnostic biomarker, comparing samples from 47 PDAC patients and 30 healthy volunteers. Plasma miRNA-221 levels were significantly higher in PDAC patients than in controls (*P* < 0.0005). In addition, plasma miRNA-221 levels were significantly reduced in postoperative samples (*P* = 0.018). Interestingly, patients affected by PDAC with high plasma concentrations of miRNA-221 reported a statistically significant correlation with metastatic and unresectable disease [53]. Komatsu et al demonstrated that miRNA-223 was significantly more expressed in PDAC tissues than in normal tissues (*p* = 0.0069). Furthermore, plasma miRNA-223 levels were significantly higher in 71 patients with PDAC than in 67 healthy individuals (*p* < 0.0001). Consequently, these plasma levels might be a useful biomarker for screening PDAC [54].

Considering the constant shedding of exfoliated cells and the production of pancreatic fluid by the tumor, feces have also been suggested as a potential biological sample in which to detect miRNAs useful as diagnostic biomarkers for PDAC. Using a subset of miRNAs that are frequently dysregulated in PDAC, Link et al found that miRNA-196a, -216a, -143 and -155, whose extraction and detection were highly reproducible, are present at lower levels in fecal samples from patients with PDAC compared to controls [55]. In another analysis, fecal miRNA-181b and miRNA-210 expression levels were significantly higher in the stools of the PDAC group compared with the normal group, highlighting their potential role as diagnostic biomarkers [56].

Mucinous cystic neoplasms of the pancreas (MCNs) are important precursors of invasive carcinoma. Cyst fluid samples could therefore be explored for early diagnosis of cancer. In this view, in a small single center-study, Farrell et al assessed the expression level of selected miRNAs by quantitative real-time-PCR in cyst fluid samples obtained endoscopically from 38 patients with MCNs. In this study miRNA-21 (*P* < 0.01) and miRNA-221 (*P* = 0.05) were expressed at significantly higher levels in malignant cysts compared with benign or premalignant cysts [57].

The main miRNAs with a potential role in PDAC in diagnosis are shown in Table 2. Even if miRNAs can be useful in the diagnosis of histological samples, a more practical use of these molecules could arise from their isolation from blood samples for early diagnostic purposes, especially in subjects at risk of developing a PDAC. Finally the study of miRNAs in the fluid of pancreatic cysts becomes useful in differentiating malignant from benign lesions.

**Table 2:** Useful miRNAs in PDAC diagnosis

miRNA	Material	Results	Reference
miRNA-148a, miRNA-217	Histological samples	Downregulated in PanIN II–III and PDAC	47
miRNA-21	Histological samples	Overexpressed in PDAC	47
miRNA-10b	Histological samples	Highly expressed in PanIN II–III	47
miRNA-122, miRNA-135b, miRNA-135b, miRNA-136, miRNA-186, miRNA-196b, MiRNA-198, miRNA-203, miRNA-222, miRNA-23a, miRNA-34c-5p, miRNA-451, miRNA-490 -3p, miRNA-492, miRNA-509-5p, miRNA-571, miRNA-614, miRNA-622, miRNA-939	Histological samples	Panel of 19 microRNAs able to discriminate pancreatic and ampullary adenocarcinomas from chronic pancreatitis and normal pancreas with high sensitivity and accuracy.	48
miRNA24, miRNA130b, miRNA-135b, miRNA-148a, miRNA-196	Histological samples	miRNA classifier able to improve PDAC diagnosis than citologyanalysys alone	49
miRNA-21, miRNA-210, miRNA-155, miRNA-196	Blood samples	Overexpressed in PDAC	50
miRNA-16, miRNA-21, miRNA-155, miRNA-181a, miRNA-181b, miRNA-196a, miRNA-210	Blood samples	Overexpressed in PDAC	51
miRNA-18a	Blood samples	Overexpressed in PDAC	52
miRNA-221	Blood samples	Overexpressed in PDAC	53
miRNA-223	Blood samples	Overexpressed in PDAC	54
miRNA-196a, miRNA -216a, miRNA-143, miRNA -155	Fecal samples	Lower levels in fecal samples from patients with PCA compared to controls	55
miR-181b, miR-210	Fecal samples	Higher expression levels in the stool of the PCa group compared with the normal group	56
miRNA-221	Cyst fluid samples	Overexpressed in malignant cysts compared with benign or premalignant cysts	57
miRNA-21	Cyst fluid samples	Overexpressed in malignant cysts compared with benign or premalignant cysts	57

## MiRNA FOR PROGNOSIS OF PDAC

Since miRNA are involved in the tumorigenesis processes, the up-regulation of onco-miRNA or the down-regulation of tumor suppressor miRNA could be used as prognostic markers in PDAC. In particular many studies have been carried out on RNA extraction of histological tissue. In a tissue microarray analysis of 80 PDAC performed using in situ hybridization, compared to samples of benign diseases, miRNA-21 expression was demonstrated in 63/80 (79%) PDACs compared to 1/12 (8%) (*p* < 0.0001) benign pancreas and 12/45 (27%) (*p* < 0.0001) CP samples [58]. Moreover, none of the benign tissues demonstrated strong miRNA-21 expression, while strong miRNA-21 expression was predictive of poorer outcome compared to absent or weak miRNA-21 expression in patients with node-negative PDAC (median OS 27.7 months vs. 15.2, *p* = 0.037).

In 2010 Grejther et al. analysed the expression levels of six miRNAs in 56 fresh frozen samples of PDAC [59]. They measured miRNA-155, miRNA-203, miRNA-210, miRNA-216, miRNA-217 and miRNA-222 by quantitative RT-PCR in a cohort of PDACs. These miRNAs were chosen because they had already demonstrated to be differentially expressed in PDAC compared to normal tissues. The relationship between miRNA expression and patient outcome showed a significant correlation between elevated miRNA expression and OS for miRNA-155 (RR = 2.50; *p* = 0.005), miRNA-203 (RR = 2.21; *p* = 0.017), miRNA-210 (RR = 2.48; *p* = 0.005) and miRNA-222 (RR = 2.05; *p* = 0.035).

MiRNA-203 was studied in 113 FFPE (Formalin Fixed, Paraffin Embedded) tissue samples of PDAC and it was associated with a higher risk of death in cases with radical resection of disease (relative risk 2.298, *P* = 0.027) [60]. Similarly, miRNA-17-5p was associated with an adverse prognosis in a review of 80 patients who underwent pancreatectomy for PDAC (*P* = 0.03) [61].

Nakata et al. published an analysis in 115 FFPE tissue samples of PDAC [62]. The analysis showed that high miRNA-10b expression was associated with the worst OS (*p* = 0.014). Jamieson et al evaluated, and subsequently validated, the correlation analysis of miRNA expression in PDAC. A cohort of 48 patients who had undergone pancreaticoduodenectomy between 2003 and 2008 was evaluated for association with tumor stage, lymph node status, and site of recurrence, in addition to OS [63]. Twenty miRNAs were associated with OS. In the initial cohort of 48 PDAC patients, high expression of miRNA-21 (HR = 3.22, 95%CI: 1.21–8.58) and reduced expression of miRNA-34a (HR = 0.15, 95%CI: 0.06–0.37) and miRNA-30d (HR = 0.30, 95% CI: 0.12–0.79) were associated with poor OS following surgery and independently of clinical covariates. In an additional validation set of 24 patients, miRNA-21 and miRNA-34a expression significantly correlated with OS (*p* = 0.031 and *p* = 0.001). Additionally, the expressions of miRNA-21 and miRNA-155 were associated with tumor stage other than a poor prognosis.

Schultz et al. evaluated the expression of miRNAs from the FFPE of 225 surgically treated patients, few of them treated with adjuvant therapy [64]. High expression of miRNA-212 and miRNA-675 and low expression of miRNA-148a, miRNA-187, and let-7g predicted short OS independently of other clinicopathological variables. A prognostic index based on the five miRNAs was calculated and, for patients with a higher PI, median OS was extremely disappointing (about 1 year). Recently Zhu et al. used RT-PCR to evaluate the expression of miRNA-218 in human PDAC cells and tissue samples [65]. They observed that low miRNA-218 expression was associated with poor tumor differentiation, advanced tumor stage, higher incidence of lymph node metastasis, and tumor recurrence. Low miRNA-218 expression was associated with lower recurrence-free survival and OS than those with high miRNA-218 expression, and multivariate analysis showed that miRNA-218 may be an independent prognostic factor. Finally, in the PDAC tissue of 109 patients, miRNA-29c expression was significantly lower in pancreatic cancer tissues compared with pair-matched adjacent paracancerous tissues, suggesting that a lower level of this miRNA is associated with a low prognosis [66].

MiRNA as prognostic biomarkers have also been evaluated in non-invasive techniques in PDAC patients. Many studies have shown that the expression level of circulating miRNA-21 could distinguish cancer patients from healthy people and predict disease outcome. In a meta-analysis by Wang et al [67], the pooled HR of higher miRNA-21 expression in circulation was 2.37 (95%CI 1.83–3.06, *P* = 0.001), which could significantly predict poorer survival in carcinomas. Importantly, subgroup analysis suggested that higher expression of miRNA-21 correlated in particular with OS in gastrointestinal cancers (HR, 5.77, 95%CI 2.65–12.52). Among the miRNA that were discovered to be associated with prognosis of PDAC, miRNA-21 was demonstrated to be associated with adverse outcome in early PDAC.

Serum miRNA levels were investigated as prognostic markers. Kong et al searched the serum of 35 PDAC in various stages of cancer to correlate eventually deregulated levels of miRNAs compared to healthy individuals or CP patients [68]. Of the seven miRNAs evaluated, three were identified as expressed in PDAC in a different way compared to control groups. MiRNA-21 allowed to distinguish PDAC patients from CP (*p* = 0.033) and healthy subjects (*p* = 0.001), whereas miRNA-155 and miRNA-196a were able to differentiate ill from healthy patients (*p* = 0.0002 and 0.010, respectively). In particular, the serum miRNA-196a level was found to have a potential prognostic significance in PDAC patients (high-level vs low-level miRNA-196a, 6.1 vs 12 months, *P* = 0.007). Komatsu demonstrated that not only are plasma miRNA-223 levels significantly reduced in postoperative samples (*p* = 0.0297), but they may also discriminate between benign IPMN and malignant IPMN (*p* = 0.0963), and the progressive extent of invasiveness between malignant IPMN and pancreatic invasive ductal carcinoma (*p* = 0.0004) becoming thus a useful predictor of the malignant potential of IPMN and the invasiveness of PDAC [54]. More recently, in order to discover biomarkers for the prognosis of PDAC, by performing a miRNA gene microarray Wang et al found 66 miRNAs differentially expressed in this cancer. Moreover, with a binary logistic regression model, they identified 8 miRNAs (miRNA 1914, miRNA-4281, miRNA-1274a, miRNA-1249, miRNA-1207-3p, miRNA-466, miRNA-1290 and miRNA-31) which could completely distinguish short-OS and long-OS patients (accuracy 100%). Furthermore, seven target genes (i.e., RET, ETS1, RHOA, NUMB, TIAM, ITGA5 and YY1) of these 8 significant miRNAs were associated with control of cell fate decisions, invasion and angiogenesis enhancement. These miRNAs and their gene targets may be potential prognostic biomarkers for PDAC [69].

The conclusion from this overview is that a correlation with prognosis exists for miRNA in PDAC. A putative miRNA is fair to be validated in the clinical setting. MiRNA-21 expression seems to be consistently associated with a bad prognosis in various series but needs to be prospectively validated. MiRNAs with a putative impact on prognosis are shown in Table 3.

**Table 3:** miRNAs impact on PDAC prognosis

miRNA	Expression status	Impact on prognosis	Reference
miRNA-21	Highexpression	worst os in node-negative PDAC	58
miRNA-155	Highexpression	worst os	59
miRNA-203, miRNA-210, miRNA-222	Highexpression	worst os	59
miRNA-203	Highexpression	worst os	60
miRNA-17-5-p	High expression	poor prognosis	61
miRNA-10b	High expression	worst os	62
miRNA-34a miRNA-30d	High expression	worst os	63
miRNA-212 miRNA-675 miRNA-let-7g	Highexpression	short os	64
miRNA-148a miRNA-187	Low levels	short os	64
miRNA-218	Low levels	short os and recurrence free interval	65
miRNA-29c	Low levels	short os	66
miRNA-21	High expression	poorer survival in general carcinomas	67
miRNA-196a	High expression	worst os	68
miRNA-223	High expression	worst os	54
miRNA 1914, miRNA-4281, miRNA-1274a, miRNA-1249, miRNA-1207-3p, miRNA-466, miRNA-1290 and miRNA-31	High expression	worst os	69

## THERAPEUTIC IMPLICATION OF MIRNA IN PDAC

Each miRNA could influence the expression of various target genes, increasing or decreasing the expression of specific molecules, and this may lead to therapeutic possibilities. However, despite this interesting perspective, critical obstacles that often involve the delivery of miRNA-targeting agents must still be overcome before transition to clinical applications. The limitations that can be overcome with the delivery include poor stability in *in vivo* biodistribution, inadequate interruption and saturation of endogenous RNA, and the unpleasant side effects, if we consider that only few pathophysiological miRNA features are known. Therefore there are numerous preclinical data but no clinical trials on the use of miRNAs in PDAC, and few clinical trials in other cancers [70].

Since the up-regulation of onco-miRNA leads to anti-apoptosis, proliferation and metastasization, antisense oligonucleotides have been developed to restore normal levels of these molecules and to reduce the proliferative activity of tumor cells. On the contrary, since the down-regulation of tumor suppressor miRNA leads to cancer spreading, ectopic expression of these molecules is able to restore the antitumor activity of their target genes. For example in the first case, Tsuda et al. discovered Gli-1-miRNA-3548 and found that its corresponding antisense oligonucleotide Duplex-3548 inhibits the proliferation of a tumor cell line of PDAC (MiaPaCa-2), retarding cell division and activating apoptosis [71–72]. In the second it was demonstrated that ectopic expression of miRNA-96 can reduce the proliferation, migration and invasion of pancreas cancer cells; this miRNA directly addresses the oncogene KRAS, and downregulates the phosphorylated Akt (P-Akt) signaling pathway [73]. Restoration of miRNA-96 in a murine model of human PDAC with subcutaneous implanted MiaPaCa cells reduced tumor growth reducing KRAS pathway activation [74], suggesting its therapeutic potential in PDAC. In literature there are more studies of ectopic expression via intratumoral or systemic delivery than antisense miRNA. The reason is the technical difficulty of constructing miRNA antisense which is not always able to pair up and counteract the activity of onco-miRNA. On the contrary, it appears relatively easy to replace oncosuppressor miRNAs. In this case the difficulty is caused by the delivery of these molecules. Currently, both viral vectors and non-viral delivery systems can be developed to circumvent these challenges. Generally viral vectors are efficient, but the toxicity and immunogenicity limit their clinical use. To bypass this problem, the non-viral delivery systems are those most investigated in biopharmaceutical studies, among these Lipid-based delivery system or polymeric nanoparticles (such as Polyethyleneimine, Dendrimers and Exosomes). These latter systems defend the oligonucleotide from digestion by nucleases, enhancing its life time and improving miRNA cellular uptake.

Let-7 and miRNA-34 are currently the most studied tumor suppressor miRNA downregulated in various solid tumors such as PDAC [75–76]. Restoring these molecules resulted in great inhibition of tumor growth, and these are the most studied miRNA in preclinical therapeutic studies. MiRNA let-7 is involved in proliferation of the K-ras depending pathway. Torrisani et al demonstrated that restoration of let-7 levels induced a reduction of K-ras expression and its related kinases, with the inhibition of mitotic processes and consequently cell proliferation in Capan-1 cells (PDAC cell line). Indeed, both intratumoral miRNA injection or xenograft of Capan-1 cells overexpressing let-7 miRNA fail to inhibit tumor PDAC subcutaneous growth in athymic mice [75]. They justified this failure in part for the molecule itself, and/or for the delivery method. In fact, restoration of this miRNA both in the form of a let-7 mimic or a viral mediated delivery resulted in significant reduction of the tumor growth xenograft model in an *in vivo* transgenic mouse model of human non-small cell lung cancer [77]. Moreover in malignant mesothelioma and lung cancer, Ephrin-A1 conjugated nanoparticles of let-7a conspicuously restrain tumor development as compared to either ephrin-A1 or let-7a nanoparticle alone [78]. No other let-7 delivery *in vivo* study is present in the current literature on PDAC.

Viral transfection of PDAC cells with miRNA-34 regulates genes codifying for cell-cycle progression, cellular proliferation, apoptosis, DNA repair, and angiogenesis, suggesting a therapeutic approach [76]. Later it was demonstrated that restoration of miRNA-34 both with miRNA-34 mimics and with infection with lentiviral miRNA-34-MIF not only improved apoptosis and reduced proliferation but also impaired tumor stem cells [79]. In fact, *in vitro* the restoration of these miRNA levels down-regulated Bcl-2 and Notch1/2 stemness genes and reduced *in vitro* the inhibition of tumor sphere (tumor stem cell colonies) formation reducing tumor growth in pancreatic cancer cell engraftment. Moreover a sensible decrease of chemo and radio-resistence was also demonstrated. In another preclinical study, the systemic delivery of miRNA-34 reassembled into a lipid-based nanovector was evaluated. In particular it has been demonstrated that the growth of subcutaneous xenograft of MiaPaCa-2 (a PDAC cell line) was reduced by intravenous administration of the miRNA nanovector [80]. Furthermore, systemic delivery of miR-34a mimics in combination with liposomial formulation has been explored in other cancers. This intravenous delivery did not induce liver and kidney failure and did not induce an immune response [81]. However in PDAC Hu et al. developed miRNA-34a-delivering nanocomplexes with a tumor-targeting and bifunctional CC9 peptide for pancreatic cancer therapy. They demonstrated that this new delivery specifically induced apoptosis and pancreatic tumor growth inhibition [82]. Recently a Phase I clinical trial (NCT01829971) has been proposed with microRNA-RX34 Liposomal Injection. In particular it is an on-going, multicenter, open-label, dose-escalation study that investigates the safety, pharmacokinetics and pharmacodynamics of this molecule involving unresectable primary liver cancer or advanced or metastatic cancer with liver involvement and hematologic malignancies [83]. This phase I clinical trial together with the study of Hu et al lays the foundations for a Phase I study in pancreatic cancer.

There are many other miRNA which have been studied in preclinical studies. MiRNA-204 is a putative regulator of myeloid cell leukemia-1 (Mcl-1), and in PDAC there are low levels of this miRNA and an overexpression of Mcl-1. Ectopic production with miRNA-204 mimic results in a decrease in Mcl-1 levels and a decrease in cell viability. [84]. Yan et al. described that miRNA-20a deregulate at the posttranscriptional level STAT3, a mediator of many genes involved in cellular processes such as cell growth and apoptosis. In this study the role of lentivirus-mediated overexpression of miRNA-20a was evaluated in two pancreatic carcinoma cell lines (BxPC-3 and Panc-1) and immortal human pancreatic duct epithelial cell line H6C7, and in subcutaneous PDAC cells injected into nude mice. They demonstrated that microRNA-20a overexpression inhibits migration and invasion, altering the cell cycle profile *in vitro* and inhibits growth and metastasis of PDAC in an *in vivo* model [85]. The miRNA-143/145 cluster was also analyzed as a potential tumor inhibitor. In fact, miRNA 143 delivery inhibited migration and invasion of Panc-1 cells *in vitro* and, on the other hand, liver metastatization and xenograft tumor growth *in vivo* [86]. Moreover, delivery of the miRNA-143/145 cluster complexed nanovector of 100 nm in diameter hindered tumor growth in MiaPaCa-2 subcutaneous and orthotopic (intrapancreatic) xenografts [87]. In an *in vivo* model targeting the metastatic pathway, Hs766t-L2 cells transfected with miRNA-29c were orthotopically implanted into male nude mice. Overexpression of miRNA-29c suppresses pancreatic cancer liver metastasis determining a key role in the PDAC metastatic process [66].

The increase of miRNA-219-1-3p in PDAC cell lines decreases cell proliferation and migration. Furthermore, in the same study an *in vivo* experiment showed that miR-219-1-3p injection in xenografted PDAC mice reduced tumor growth through MUC4 oncomucin reduction [41]. MiRNA-203 transfection suppresses CFPAC-1 proliferation through apoptosis and cell cycle arrest. Moreover, in a female BALB/cA-nu nude mice model, the overexpression of miRNA-203 inhibited tumor growth [42]. More recently, Cioffi et al injected PDAC cells overexpressing miR-17-92 into female NU-Foxn1nu nude mice treated with G. The authors showed a reduction of *in vivo* tumourigenicity, indicating that high levels of miRNA-17-92 forced CSCs into a more differentiated state, reducing their self-renewal capacity *in vivo* and sensitizing them to G [45]. Although, as it has been seen, oncomiRNAs are difficult to study from the therapeutic point of view, there are significant approaches for miRNA-21, the most studied of miRNAs in oncogenesis. In an *in vitro* study, this miRNA modulated the biological functions of pancreatic cancer cells including their proliferation, invasion, and chemoresistance [88]. Sicard et al demonstrated that targeting oncogenic miRNA-21 with a lentiviral transported antimiRNA strongly inhibits Mia-Pa-Ca2 pancreatic cancer proliferation and tumor growth in a nude mice engraftment *in vivo*; moreover he demonstrated that miRNA-21 antagonist enhances tumor angiogenesis, improving the flow of drugs and overcoming chemoresistance. In fact, gemcitabine and miRNA-21 are synergistic and this cotreatment led to a very important antitumoral effect that is rarely achieved in this experimental model [89]. However, other authors have demonstrated that the inhibition of more oncomiRNA has a better synergistic effect. Park and colleagues demonstrated that antisense oligonucleotides directed toward miRNA-21 and miRNA-221 sensitized the effects of G, and that the antisense miRNA-21 + G combinations were synergistic, confirming the role of miRNA-21 in modulating the response to cytotoxic drugs *in vitro*. Furthermore, the inhibition of these mi-RNAs causes a reduction in cell growth inhibition via activation of gene PTEN, RECK and p27, proteins involved in the control of cell proliferation [90]. Giovanetti et al. obtained similar results with transfection with antisense miRNA-21 in terms of a decrease of antiproliferative effects and apoptosis induction by G [91]. More recently Zhao Y et al showed that in L3.6pl, a highly metastatic human PDAC cell line, miRNA-21 and miRNA-221 antisense oligonucleotide transfection L3.6pl, reduces cell proliferation, invasion, and gemcitabine and 5-Fluorouracil chemoresistance. In an *in vivo* experiment the authors documented that the combination of miRNA-21 and miRNA-221 antisense oligonucleotides reduced primary tumor growth and metastasis compared to single anti-miRNA treatment [92]. The synergic effect of inhibition of onco-miRNAs has been documented by Frampton et al. They analyzed the combined effects of up-regulated miRNAs in PDAC cell lines and using PDAC xenografts grown in nude mice. In particular they found that the cooperative action of these 3 miRNAs, miRNA-21, miRNA-23a, and miRNA-27a, inhibits some tumor suppressor molecules such as PDCD4, BTG2, and NEDD4L. Inhibition of miRNA-21, miRNA-23a, and miRNA-27a had synergistic effects in reducing proliferation and growth of PDAC cells both *in vivo* and *in vitro*. The inhibition level was greater than inhibition of miRNA-21 alone [93]. In an *in vitro* study, the inhibition of miR-27a reduced growth, colony formation and migration of PANC-1 and MIA PaCa-2 [29].

In addition, in a particular *in vitro* study of miRNA-31, it was shown that the same miRNA is overexpressed in ASPC-1 and HPAF-II and reduced in MIA PaCa-2; three PDAC cell lines. It has also been seen that inhibition of miRNA-31 in HPAF-II reduces the proliferation, migration and invasiveness of these cells. Similar results are achieved with the delivery of miRNA-31 in MIA PaCa-2. Interestingly, in ASPC-1 both inhibition and delivery of the miRNA resulted in a reduction of the proliferative and metastatic, which show how little is known about miRNAs and how they have a different action depending on the cellular phenotype [94].

In conclusion, reduction or suppression of miRNAs that function as a tumor suppressor gene leads to the formation of tumors, the use of exogenous miRNA in the PDAC and the re-establishment of their normal levels could result in inhibition of tumor growth. Moreover, several studies have evidenced that other miRNAs act as oncogenes, and cancers may develop due to oncogenic miRNA overexpression, which holds a promise for the therapeutic use of miRNA inhibitors. Although we still have a way to go in identifying key miRNAs of clinical importance in PDAC, the rapid development of the field is likely to advance many preclinical projects to humans in the near future. Table 4 summarizes these preclinical studies in PDAC, which have led to a new therapeutic approach and provided the first data on the therapeutic role of miRNAs. However, the delivery, the pharmacokinetics, pharmacodynamics and possible toxicity that these molecules may give are obstacles that must be overcome before moving on to a clinical phase.

**Table 4:** Summary of preclinical studied of miRNA role in PDAC therapy

miRNA	Mechanisms of action	Type of study	Results	Reference
Gli-1-miRNA3548	Use of antisense oligonucleotide	*In vitro* study in MiaPaCa-2	Inhibition of proliferation and cell division Activation of apoptosis	72
miRNA96	Ectopic expression of this miRNA	*In vitro* study in MIA PaCa-2, PANC-1, and BxPC-3	Reduction of the proliferation, migration and invasion	73
miRNA96	Ectopic delivery of this miRNA	Murin model of human PDAC with subcutaneous implanted MiaPaCa cells	Reduced tumor growth, reducing KRAS pathway activation	74
Let-7	Ectopic expression of this miRNA	*In vitro* study in Capan-1 cells	Reduction of proliferation of K-ras depending pathway	75
miRNA34	Viral transfection with miRNA	study of pancreatic cancer cells	Regulation of genes codifying for cell-cycle progression, cellular proliferation, apoptosis, DNA repair, and angiogenesis	76
miRNA34	restoration of miRNA both with miR-34 mimics or infection with lentiviral miR-34-MIF	*In vitro* study and tumor pancreatic cancer cell engraftment.	Improvement of apoptosis. Reduction of proliferation, Reduction of tumor stem cells decreasing chemio and radio-resistance	79
miR-34a	Systemic delivery of miRNA-34 into a lipid-based nanovector	In particular it has been demonstrated that the growth of subcutaneous xenograft of MiaPaCa-2 (a PDAC cell line)	Inhibition of pancreatic cancer growth	80
miRNA34	miR-34a-delivering nanocomplexes with a tumor-targeting and bifunctional CC9 peptide	Athymic female mice (BALB/c strain) subcutaneously injected with PANC-1 cells.	Specific induction of apoptosis and pancreatic tumor growth inhibition	82
miRNA204	Ectopic production with miR-204 mimic	Three de-identified human tumors implanted subcutaneously into SCID animals	Decrease in Mcl-1 levels and a decrease in cell viability	84
miR-20a	lentivirus-mediated overexpression of microRNA-20a	*In vitro* study on 2 PDAC (BxPC-3 and Panc-1) and immortal human pancreatic duct epithelial cell line H6C7, and in subcutaneously PDAC cell injected into nude mouse	Inhibition of migration and invasion alteringcell cycle profile vitro and inhibits growth and metastasis of PDAC *in vivo* model	85
miR-143/145	miRNA delivery	*in vivo* xenograft of PDAC tumor	Inhibition of migration and invasion of Panc-1 cells *in vitro* and, on the other hand, liver metastasization	86
miR-143/145	miR-143/145 cluster complexed nanovector of 100 nm diameter	MiaPaCa-2 subcutaneous and orthotopic (intrapancreatic) xenografts	Reduction of tumor growth	87
miRNA-29c	miRNA-29c cell transfection	Hs766t-L2 cells transfected with miRNA-29c orthotopically implanted into male nude mice	Reduction of liver metastasis	66
miR-219-1-3p	miRNA delivery	miR-219-1-3p injection in xenografted PDAC mice	Reduction of tumor growth through MUC4 oncomucin reduction	41
miRNA-203	MiRNA-203 transfection	*In vitro* study on CFPAC-1 and engraftment into female BALB/cA-nu nude mice model	Suppression of proliferation through apoptosis and cell cycle arrest. Inhibition of tumor growth *in vivo*	42
miR-17-92 cluster	PDAC cells overexpressing miR-17-92 sensitising them to G	Engraftment into female NU-Foxn1nu nude mice treated with G.	reduction of *in vivo* tumourigenicity, forcing CSCs into a more differentiated state, reducing their self-renewal capacity *in vivo*	45
miRNA21	lentiviral transported antimiRNA	*In vitro* study on MiaPaCa2 and nude mice engrafment *in vivo*	Inhibition of proliferation and tumor growthenhances tumor angiogenesis, improving the flow of drugs overcoming chemoresistance Synergistic effect of gemcitabine and miR-21	88–89
miR-21/221	antisense oligonucleotides directed toward miR-21 and miR-221	*In vitro* on PDAC cell line	sensitized the effects of G, and the antisense miR-21 + G combinations were synergistic	90
miR-21/221	inhibition of these mi-RNAs	*In vitro* on PDAC cell line	Reduction in cell growth inhibition via activation of gene PTEN, RECK and p27, proteins involved in the control of cell proliferation	90
miRNA 21	transfection with antisense miR-21	*In vitro* study on seven PDAC cell lines and seven primary PDAC cultures	Decrease of antiproliferative effects and apoptosis induction by G	91
miRNA-21 and miRNA-221	miRNA-21 and miRNA-221 antisense oligonucleotides transfection	*In vitro* in L3.6pl, a highly metastatic human PDAC cell line	Reduction of cell proliferation, invasion, and gemcitabine and 5-Fluorouracil chemoresistance	92
miR-21, miR-23a, miR-27a	inhibition of onco-miRNAs	*In vitro* with PDAC cell lines and using PDAC xenografts grown in nude mice	Synergic effect of inhibition of onco-miRNAsmiR-21, miR-23a, and miR-27a	93
miR-27a	inhibition of onco-miRNAs	PANC-1 and MIA PaCa-2	Reduction of cell growth, colony formation and migration	30
miRNA-31	Duble function miRNA	The same miRNA is overexpressed in ASPC-1 and HPAF-II and reduced in MIA PaCa-2, three PDAC cell lines.	Inhibition of miRNA31 in HPAF-II and in MIA PaCa-2 reduces the proliferation, migration and invasiveness of these cells.In the ASPC-1 both inhibition and delivery of the miRNA result in a reduction of the proliferative and metastatic phenotype.	94

## CONCLUSION: CURRENT AND FUTURE APPLICATIONS OF MiRNA IN PDAC

All the above-mentioned studies strongly highlight the potential usefulness of miRNAs in PDAC tumorigenesis, diagnosis, prognosis, and therapy. The diagnostic role of miRNA represents probably the most attractive and up-to-date way of studying these molecules in oncology. Recently, a metanalysis of 18 studies showed that the use of miRNAs has a potential diagnostic value with a relatively high sensitivity and specificity for PDAC; in particular, the use of multiple miRNAs allows for discrimination of PDAC patients from healthy ones [95]. Obviously cancer research in this field needs a prospective demonstration of efficacy on a large scale through randomized prospective trials. A recent case control study showed that two diagnostic panels based on miRNA expression in blood have the potential (alone and with CA 19.9) to distinguish patients with pancreatic cancer from healthy controls [96]. Prospective research is necessary to understand if there could be a clinical application in the early detection of PDAC. Even if the clinical use of miRNAs as predictive/prognostic markers is very close, the use of miRNA or miRNA mimetic antagonists in therapy appears more distant, because we need firstly to perfect the sequences and their delivery, and especially to understand what are the real physiological functions of these molecules in order to cause as few side effects as possible. Currently there are several clinical trials using these new nanotechnologies in various cardiovascular diseases [97], chronic obstructive pulmonary disease [98], neurodegenerative diseases [99] and, as we have seen, some trials are investigating them in the cancer field. In particular there are some trials on the use of miRNAs in diagnosis [100], as predictive markers of toxicity [101], although those mentioned are just some of the many ongoing trials. From the therapeutic point of view there is just one study on the use of therapeutic miRNA: miRNA-34 [83]. Although we are getting closer to the first clinical trials in PDAC, we are still far from their clinical use.

In conclusion, validated and prospective systematic evaluation of this new research merits development into the PDAC setting as well as for other malignancies. The perspective is that the application of preclinical and biological models into the clinic and bedside will offer patients suffering from PDAC a new hope of cure.
